# Enhancing cortical network-level participation coefficient as a potential mechanism for transfer in cognitive training in aMCI

**DOI:** 10.1016/j.neuroimage.2022.119124

**Published:** 2022-03-21

**Authors:** Quanjing Chen, Adam Turnbull, Martin Cole, Zhengwu Zhang, Feng V. Lin

**Affiliations:** a CogT Lab, Department of Psychiatry and Behavioral Sciences, Stanford University, United States; b School of Nursing, University of Rochester, United States; c Department of Biostatics and Computational Biology, University of Rochester, United States; d Department of Statistics and Operations Research, UNC-Chapel Hill, United States; e The Wu Tsai Neuroscience Institute, Stanford University, University of Rochester, United States

**Keywords:** Cognitive training, Transfer effect, Integration, Graph theory, Participation coefficient

## Abstract

Effective cognitive training must improve cognition beyond the trained domain (show a transfer effect) and be applicable to dementia-risk populations, e.g., amnesic mild cognitive impairment (aMCI). Theories suggest training should target processes that 1) show robust engagement, 2) are domain-general, and 3) reflect long-lasting changes in brain organization. Brain regions that connect to many different networks (i.e., show high participation coefficient; PC) are known to support integration. This capacity is 1) relatively preserved in aMCI, 2) required across a wide range of cognitive domains, and 3) trait-like. In 49 individuals with aMCI that completed a 6-week visual speed of processing training (VSOP) and 28 active controls, enhancement in PC was significantly more related to transfer to working memory at global and network levels in VSOP compared to controls, particularly in networks with many high-PC nodes. This suggests that enhancing brain integration may provide a target for developing effective cognitive training.

## Introduction

Cognitive training is seeing growing use in populations with diverse cognitive abilities (Hill et al., 2017; Lampit et al., 2014; Tang et al., 2019). Improvements in the trained domain have been demonstrated robustly in older adults for whom the aim is to prevent or slow cognitive decline, especially in the context of dementia (Gates et al., 2019). However, for cognitive training to be maximally clinically useful, it needs to result in benefits to cognition more broadly beyond the trained domain: i.e., to show a transfer effect, and to be feasible in individuals at higher risk for dementia who may already be showing signs of cognitive decline. It has been proposed that transfer relies on the training of processes that are common across trained and untrained domains resulting in changes in brain architecture that go beyond purely task activation patterns (Lovden et al., 2011). However, empirical findings suggest that these common processes need to show robust engagement, which is often absent in older adults and those at-risk for dementia (Dahlin et al., 2008), to result in transfer. Taken together, these findings suggest that, to have maximum clinical utility, cognitive training programs should target neural processes that 1) show robust engagement in those at-risk for dementia, 2) are domain-general across a range of cognitive processes, 3) reflect sustained changes in brain architecture. Identifying neural processes that meet these criteria is a key step in understanding how best to develop training programs that will cause transfer in individuals at-risk for dementia.

A key at-risk group is older adults with amnestic mild cognitive impairment (aMCI), who have a significantly elevated risk of progression to Alzheimer’s disease (AD) (Sperling et al., 2011), show signs of impairment predominantly in memory-related processes (Hirni et al., 2013), and demonstrate pathology in memory-related regions (Braak et al., 2000; Palmqvist et al., 2017; Vogel et al., 2020). Studies have shown significant differences in how older adults with aMCI engage cognitive processes (Li et al., 2015), with important implications for cognitive training. Regions engaged during visual attention (i.e., those in visual and attention networks) appear to be the least functionally different in aMCI, suggesting they may represent a potential target for effective cognitive training. Tasks that rely on Processing Speed and Attention (PS/A) are known to activate these regions, suggesting they meet the first criteria for effective cognitive training in aMCI. However, while training using these tasks has been shown to improve trained domains in individuals with aMCI, there is limited evidence of transfer to memory-related domains (Miotto et al., 2018; Rosen et al., 2011). Directly analyzing sustained, domain-general brain mechanisms responsible for transfer in specific individuals following training using these tasks may provide a means of determining how to enhance transfer effects in the population as a whole.

Recently there has been a shift towards understanding neural function in terms of brain topology rather than analyzing specific regions in isolation. Research has shown that an individual’s brain topology shows trait-like properties (i.e., is reliable over time and individual-specific) (Finn et al., 2015; Gratton et al., 2018), and relates robustly to their cognitive traits, including in older adults at various stages of cognitive decline (Avery et al., 2020). Brain topology therefore reflects a key target for the identification of long-lasting brain mechanisms that can be exploited to improve transfer effects of cognitive training in individuals with aMCI. One potential mechanism involves the capacity of the cerebral cortex for integrating information across a range of cognitive processes via connections that link functionally specialized networks. Recent research suggests this capacity can be understood by studying Participation Coefficient (PC; how divese a brain region’s connections are to different functionally specialized networks) and that cortical networks containing many high PC nodes are particularly important for supporting the integration of information (Bertolero et al., 2017). Cortical networks that include many high PC nodes could facilitate effective transfer of training using PS/A tasks because 1) these networks are robustly activated by PS/A tasks in aMCI (Li et al., 2015), 2) high PC nodes in these networks are critical for a wide range of cognitive processes (Warren et al., 2014), and 3) PC reflects a property of brain architecture with relatively high test-retest reliability, suggesting changes may persist over time (Du et al., 2015). Importantly, preliminary research suggests that training using PS/A tasks causes an increase in functional connectivity between regions within these networks (Ross et al., 2019) in healthy older adults, suggesting the potential for improving PC in these regions via tasks involving PS/A.

Using a randomized controlled trial design, we examined the underlying brain mechanisms of transfer effects by looking at cortical network-level PC in participants with aMCI that completed a 6-week VSOP (vision-based speed of processing) cognitive training that is vision-driven PS/A-oriented, in comparison to an active control group engaging in computerized mental leisure activities (MLA) of Sudoku, cross-word puzzles, and solitaire. We collected behavioral data on trained (Useful Field-of-View; UFOV) and transferred cognitive domains (working memory and episodic memory), as well as brain imaging data on resting-state and a PS/A task related fMRI immedilatey before and after 6-week intervention. We hypothesized that transfer to untrained domains in older adults with aMCI would relate to increased network PC in specific networks including large numbers of high PC nodes that are robustly activated by VSOP training. By assessing the interaction of group-by-neural change, we wanted to assess whether enhanced network PC in the intervention group specifically was related to increased transfer effects. We expected to see the effects of network PC particularly in networks with high PC nodes that are activated by VSOP training, such as ventral and dorsal attention, and frontoparietal networks (Bertolero et al., 2017). We also tested whether changes of network PC mediated the relationship between trained and untrained domains.

## Results

**Fig. 1.**
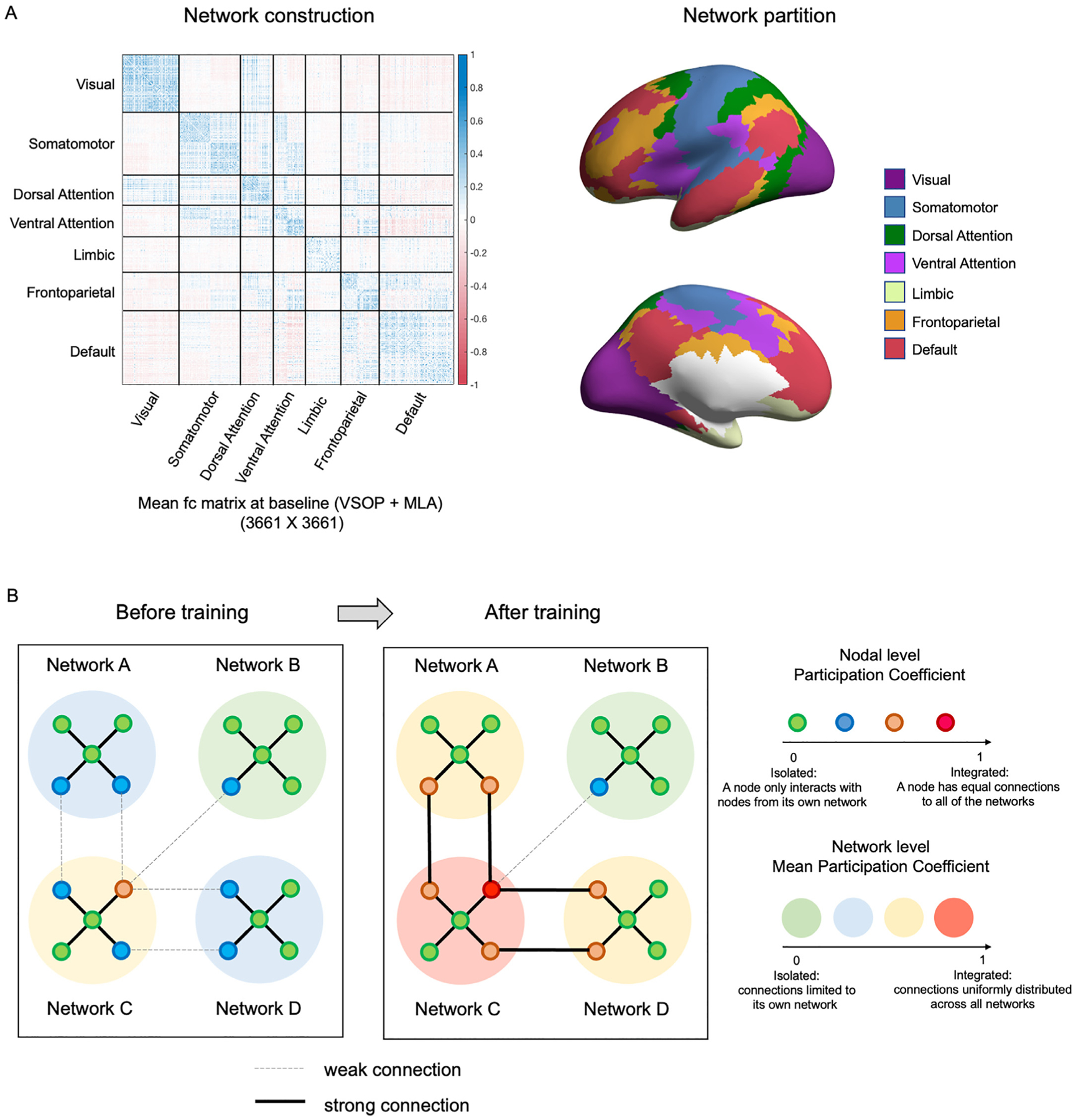
**A.** For each participant, we generated a functional connectivity matrix based on 3661 cortical nodes with Freesurfer recon-all parcellation for baseline and post-intervention respectively. Then each node was assigned to one of the seven large-scale functional networks defined by Yeo et al. (2011). Left is an example of mean functional connectivity matrix for the whole group at baseline. Right shows the networks defined by Yeo. **B.** Schematic overview of network-level Participation Coefficient (PC) analysis. We first calculated PC for each node, which reflects how strongly a node within a given network is connected to other networks. Then we calculated network-level PC by averaging PC across nodes within each network, which quantifies the extent to which a network connects to other networks. We expected that transfer effects would relate to increased PC across large-scale brain networks after training, particularly in networks with more high PC nodes (e.g., network C).

By capitalizing on a vision-driven PS/A-oriented 6-week intervention in 84 older adults with aMCI and applying whole-brain graph theoretical methods to functional connectivity data acquired using resting state fMRI, we examined the underlying neural mechanisms of transfer effects induced by cognitive training. We randomly assigned participants into VSOP intervention and MLA control groups at a 2:1 ratio. Sample characteristics are in Table 1. There were no significant group differences in baseline characteristics. To study the intervention effect, we only included participants who had both baseline assessment and assessment immediately after the intervention period (VSOP: *n* = 49; MLA: *n* = 28). For each individual the T1 image was parcellated into 3661 network nodes using FreeSurfer and the SBCI pipeline (Cole et al., 2021) and then a 3661 by 3661 functional connectivity matrix was generated for baseline and post-intervention respectively. Fig. 1A left shows an example of the mean functional connectivity matrix for the whole group at baseline. We assigned each node to one of the seven large-scale functional networks defined by Yeo et al. (2011) (Fig. 1A right). To assess the sparsity of the functional networks, we calculated the density as the ratio of the number of positive fc edges with respect to the maximum possible edges for each participant at baseline. Then we averaged density across all participants to obtain the group mean density. The group mean density is 49% for the whole brain, 42% – 62% for between-networks, and 63% – 80% for within-networks (Supplemental Figure 1). Using these pre-defined networks, we calculated PC: nodes with functional connectivity to only nodes from their own network have a PC of 0, while nodes with many distributed between-network connections would have a PC closer to 1. We measured network PC by calculating mean PC across all nodes within a network and similarly averaged across all nodes for global analyses (see Fig. 1B for a schematic depiction). We hypothesized that transfer effects would relate to increased PC across large-scale brain networks after training, particularly in networks with more high-PC nodes (e.g., network C in Fig. 1B).

### Training effect on trained and untrained cognitive domains

**Fig. 2.**
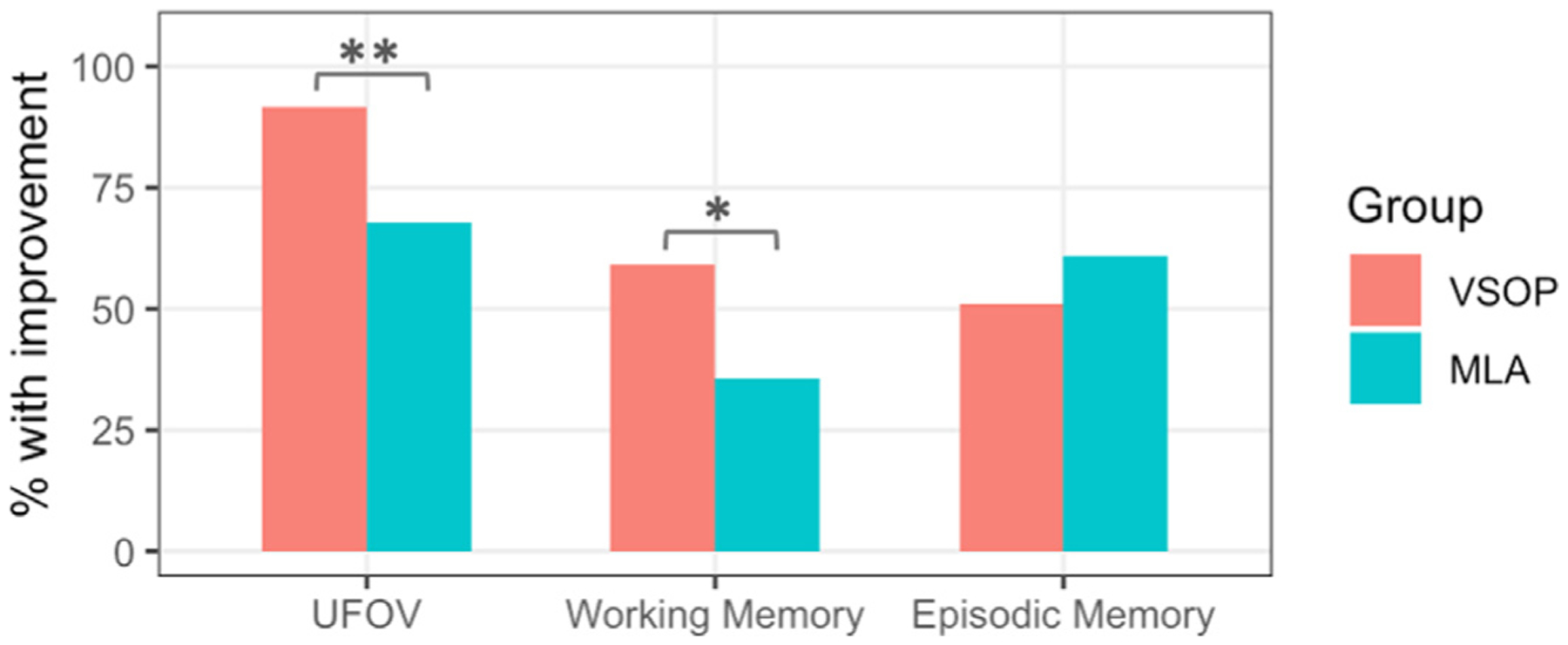
Effects of VSOP training on trained and untrained domains. Percent of participants showing reliable improvement for trained (i.e., UFOV) and untrained (i.e., working memory and episodic memory) domains for VSOP (red) and MLA (blue). * represents significant between-group difference in% with reliable improvement (*p* < 0.05).

To test whether VSOP training resulted in improvements in trained (UFOV) and untrained domains (working memory and episodic memory), we calculated the group difference in reliable improvements, where a participant was classified as having improved reliably if their performance at a follow-up occasion exceeded baseline performance by 1 SEM of baseline data (cf., ACTIVE trial (Ball et al., 2002)) (Fig. 2, see Method section for details). Significant between-group differences that were higher in the VSOP group relative to MLA included: UFOV from baseline to post-test (*g* = 0.51, *χ*^2^ = 7.07, *p* = 0.008), working memory from baseline to post-test (*g* = 0.22, *χ*^2^ = 3.93, *p* = 0.048). These results suggest that VSOP training leads to improvements in working memory. There was no significant effect of VSOP training on episodic memory.

### Baseline measure of task activation and PC

**Fig. 3.**
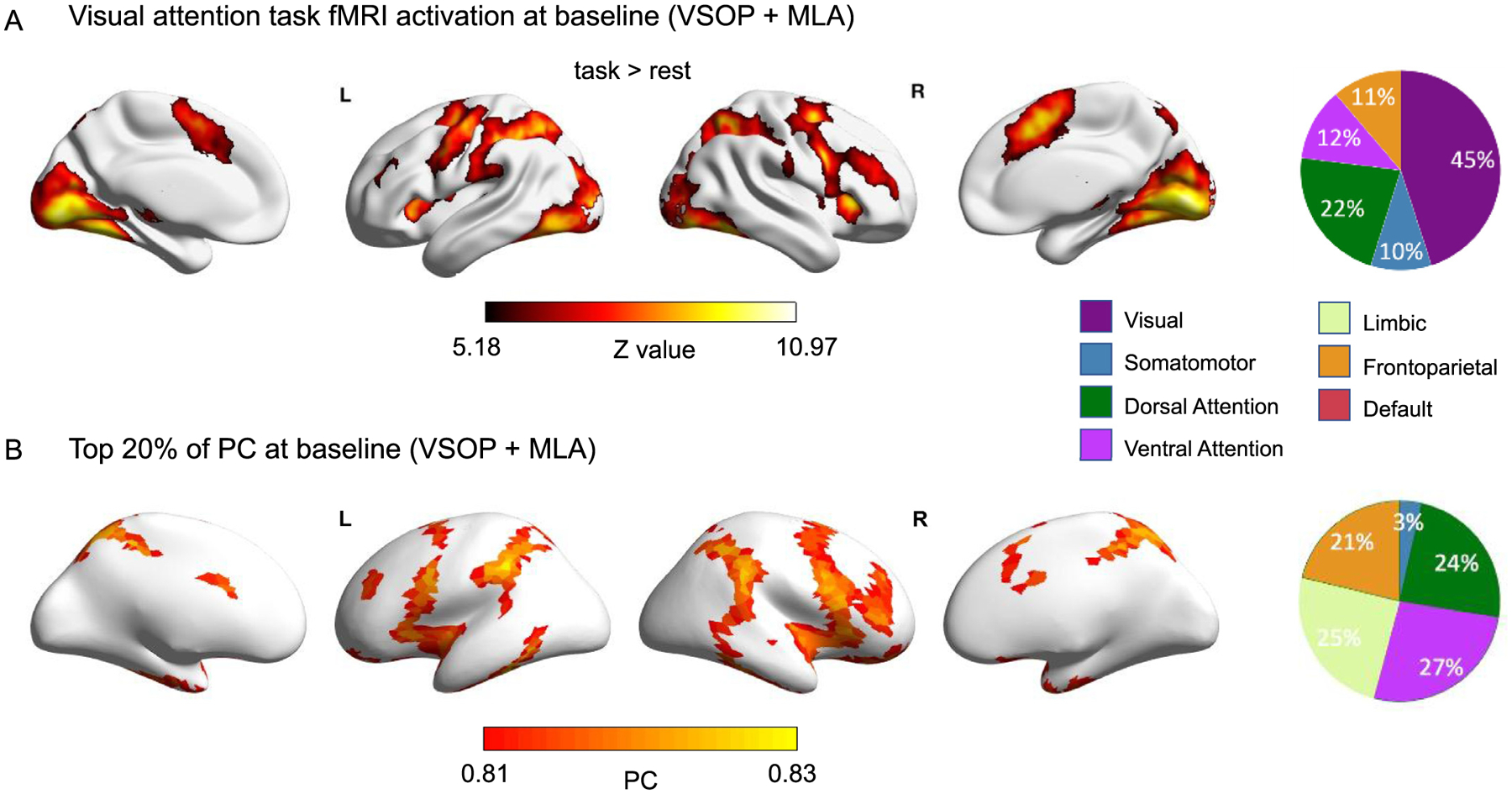
**A.** Task activation for the whole group at baseline. For the visual attention task fMRI, we computed the group-level BOLD contrast map showing stronger activity for task stimuli compared with fixation periods, FDR corrected *p <* 0.05. **B.** Top 20% of PC for the whole group at baseline. The pie charts were divided into different segments showing percentages of voxels or nodes within each network.

To confirm that PS/A tasks similar to those used in our cognitive training program robustly activate regions in networks that include high-PC nodes in aMCI, we measured brain activity during a visual attention task across both groups at baseline and calculated percentages of significant voxels within each of the 7 Yeo networks (Fig. 3A). In addition to unimodal sensory networks (visual and sensorimotor), results showed that the visual attention task activated regions in higher-order dorsal attention (DAN), frontoparietal control, and ventral attention networks (VAN) including important high-PC nodes (Bertolero et al., 2017) such as the insula (VAN), dorsal anterior cingulate cortex (VAN), dorsolateral pre-frontal cortex (FPCN), and superior parietal cortex (DAN) (Fig. 3A). This suggests that performance on this task robustly engages high-PC networks in aMCI, in line with studies suggesting they are relatively preserved in these individuals (Li et al., 2015). Previously studies suggest that while diverse club connectivity is relatively spared in aMCI, certain topological properties of these regions may show differences to healthy older adults (Xue et al., 2020). To assess the spatial pattern of high-PC nodes in individuals with aMCI, we plotted the top 20% nodal PC (high-PC nodes, Bertolero et al. (2017)) across both groups at baseline (Fig. 3B). In line with studies in younger adults, we found highest numbers of high-PC nodes in the limbic, frontoparietal, and both attention networks, and lowest in the visual and default mode networks, suggesting preservation of PC distribution in aMCI (Bertolero et al., 2017), and confirming the importance of the frontoparietal and attention networks that are robustly activated by visual attention tasks in our sample for integration across multiple networks. To assess the extent to which PS/A tasks similar to those used during training activated high-PC nodes defined using our sample, we calculated the percentage of high-PC nodes that were significantly active during the PS/A task, and found that 51% of these nodes were significantly active. This suggests that these tasks are relatively well-suited to engage these nodes, although future research may be needed to identify ways of improving these tasks so that they activate a greater proportion of high-PC nodes.

### Relationship of brain PC with VSOP-induced trained and transferred effects

To test our hypothesis that increased PC related to greater improvement in the untrained working memory domain in the intervention group (i.e., VSOP) compared to the control group (i.e., MLA), we conducted GLM models (Change of *Cognition* = *β*_0_ + *β*_1_ *Group* + *β*_2_ Change of *PC* + *β*_4_ *Change of PC* × *Group* + ԑ) at global and network levels. We expected to see significant interaction effects for Change of *PC* × *Group*.

**Fig. 4.**
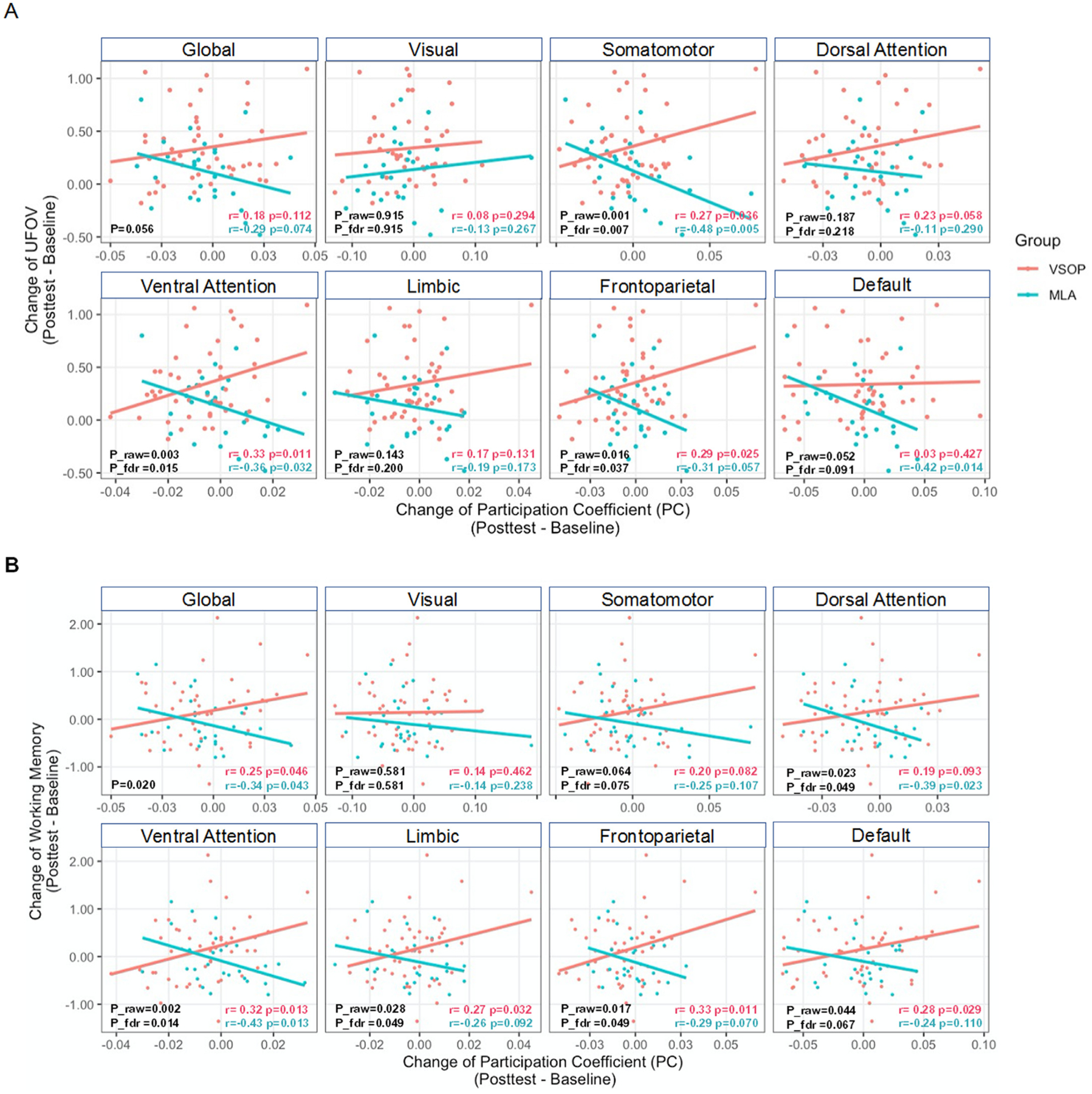
Relationship of global and network PC with VSOP-induced trained (**A**) and transferred (**B**) effects. The x-axis is the change of PC and the y-axis is the change of UFOV (trained effect) or working memory (transferred effect). Significant between-group difference was based on the interaction of change of *PC* × *Group*. To control the False Discovery Rate (FDR), p-values for the interaction effect were corrected across 7 networks using Benjamini–Hochberg (BH) procedure and both raw and FDR-adjusted p-values were reported. Pearson correlation analysis were conducted for VSOP (red) and MLA (blue) separately and raw p-values were reported.

For the trained domain, relative to MLA, greater improvement in UFOV was weakly associated with greater increases in global PC score following VSOP training (*B* = 7.80, SE = 4.08, Wald’s *χ*^2^ = 3.66, p = 0.056). When examining the effect related to network PC scores, the somatomotor network (*B* = 9.91, SE = 3.09, Wald’s *χ*^2^ = 10.32, raw *p* = 0.001, FDR-adjusted p = 0.007), ventral attention (*B* = 15.51, SE = 5.19, Wald’s *χ*^2^ = 8.95, raw *p* = 0.003, FDR-adjusted p = 0.015) and frontoparietal (*B* = 12.28, SE = 5.12, Wald’s *χ*^2^ = 5.77, raw *p* = 0.016, FDR-adjusted p = 0.037) networks shows significant relationships to trained-domain improvements after FDR correction (Fig. 4A). To additionally ensure these results were not driven by changes in head motion that are known to affect resting state FC-associated measures, we conducted paired t-tests and found no significant change in any of the six head motion parameters in either the whole sample or training group. We also included participant change in the six head motion parameters from pre- to post-training as covariates in these analyses. Including motion did not alter these results, with the same networks surviving FDR-correction, although the relationship between change in global PC and UFOV improvements was significant after including head motion parameters (*B* = 8.83, SE = 4.13, Wald’s *χ*^2^ = 4.56, *p* = 0.033).

In line with our hypothesis that increased PC relates to greater transfer effects of cognitive intervention, compared to MLA, greater improvement in working memory was associated with enhanced global PC score following VSOP training (*B* = 17.91, SE = 5.07, Wald’s *χ*^2^ = 7.71, p = 0.020). When examining the effect related to network PC scores, the strongest effect was observed in the ventral attention network (*B* = 30.88, SE = 8.05, Wald’s *χ*^2^ = 9.70, raw *p* = 0.002, FDR-adjusted p = 0.014), with dorsal attention (*B* = 21.87, SE = 9.61, Wald’s *χ*^2^ = 5.17, raw *p* = 0.023, FDR-adjusted p = 0.049), frontoparietal (*B* = 23.27, SE = 9.71, Wald’s *χ*^2^ = 5.74, raw *p* = 0.017, FDR-adjusted p = 0.049), and limbic (*B* = 24.17, SE = 11.00, Wald’s *χ*^2^ = 4.84, raw *p* = 0.028, FDR-adjusted p = 0.049) networks being significant after FDR correction as well (Fig. 4B). With change of head motion parameters as covariates, the ventral attention network was still the most strongly related to improvements in working memory (*B* = 31.17, SE = 9.73, Wald’s *χ*^2^ = 10.26, raw *p* = 0.001, FDR-adjusted p = 0.007), however, the dorsal attention (*B* = 21.64, SE = 9.92, Wald’s *χ*^2^ = 4.75, raw *p* = 0.029, FDR-adjusted *p* = 0.068), frontoparietal (*B* = 22.39, SE = 9.55, Wald’s *χ*^2^ = 5.49, raw *p* = 0.019, FDR-adjusted *p* = 0.067), and limbic (*B* = 21.28, SE = 10.91, Wald’s *χ*^2^ = 3.81, raw *p* = 0.051, FDR-adjusted *p* = 0.089) networks no longer passed FDR-correction. Given the relatively marginal p-values in these relationships and the change following the addition of motion covariates, we suggest particular caution should be used when interpreting these findings outside of the ventral attention network.

There were no significant effects of increased PC following VSOP training on episodic memory.

### Main effect of training on PC

**Fig. 5.**
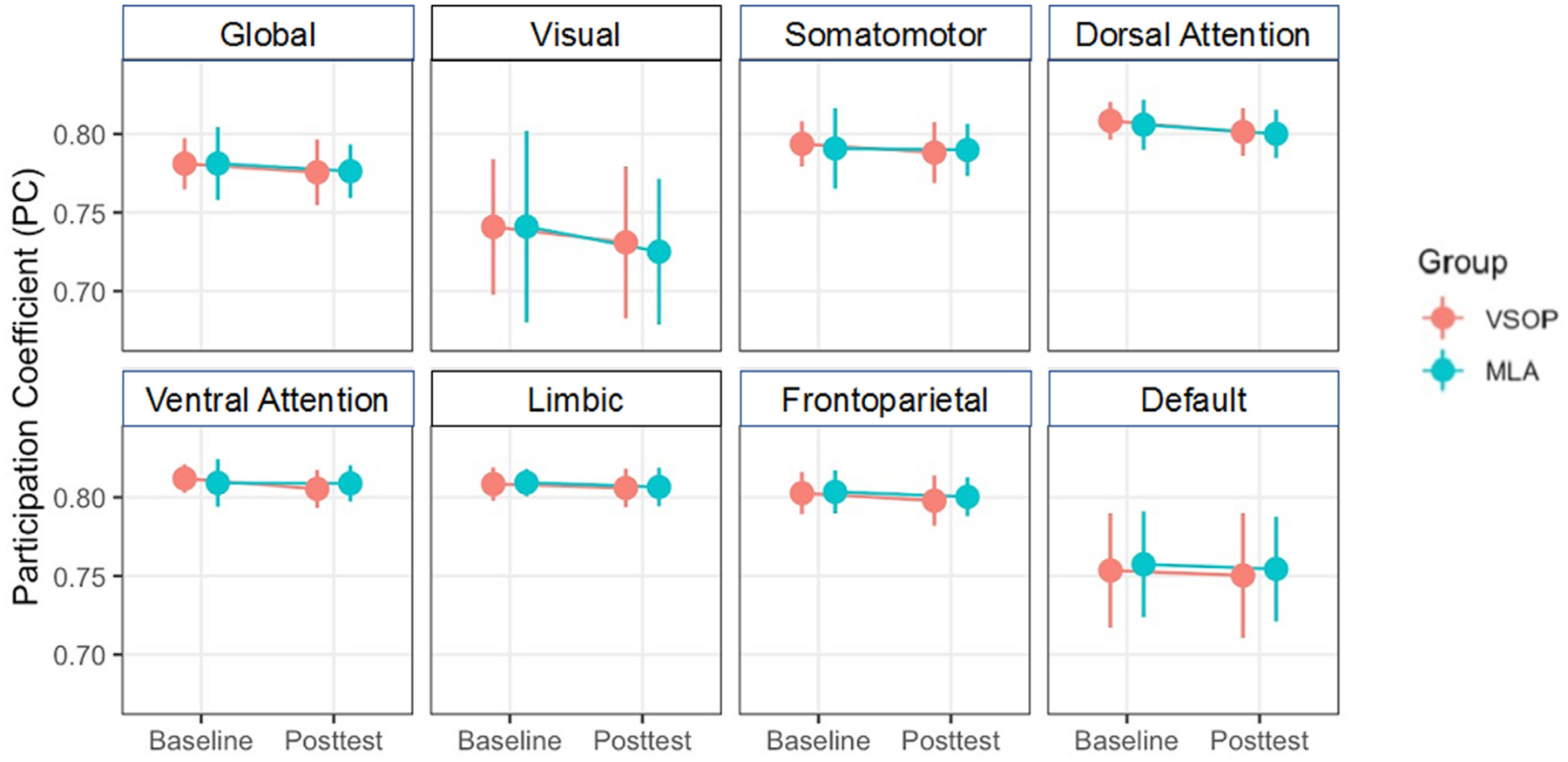
Effects of VSOP training on global and network PC. We didn’t find any significant group-by-visit comparison of global or network PC. Error bars represent standard error of the mean.

To test whether VSOP training resulted in enhancement in PC in general across participants, we performed between-group analyses with a GEE model (*PC* = *β*_*0*_ + *β*_*1*_ *Visit* + *β*_*2*_ *Group* + *β*_*3*_ *Visit* × *Group* + ԑ) (Fig. 5). We didn’t find any significant interaction of *Visit* × *Group* on PC scores at global or network levels. This suggests that while individuals that showed increased PC at global and network levels following training were significantly more likely to demonstrate a transfer effect, in general the training task did not lead to an increase in PC at either global or network levels.

### PC of ventral attention network mediates transfer effect in VSOP group

**Fig. 6.**
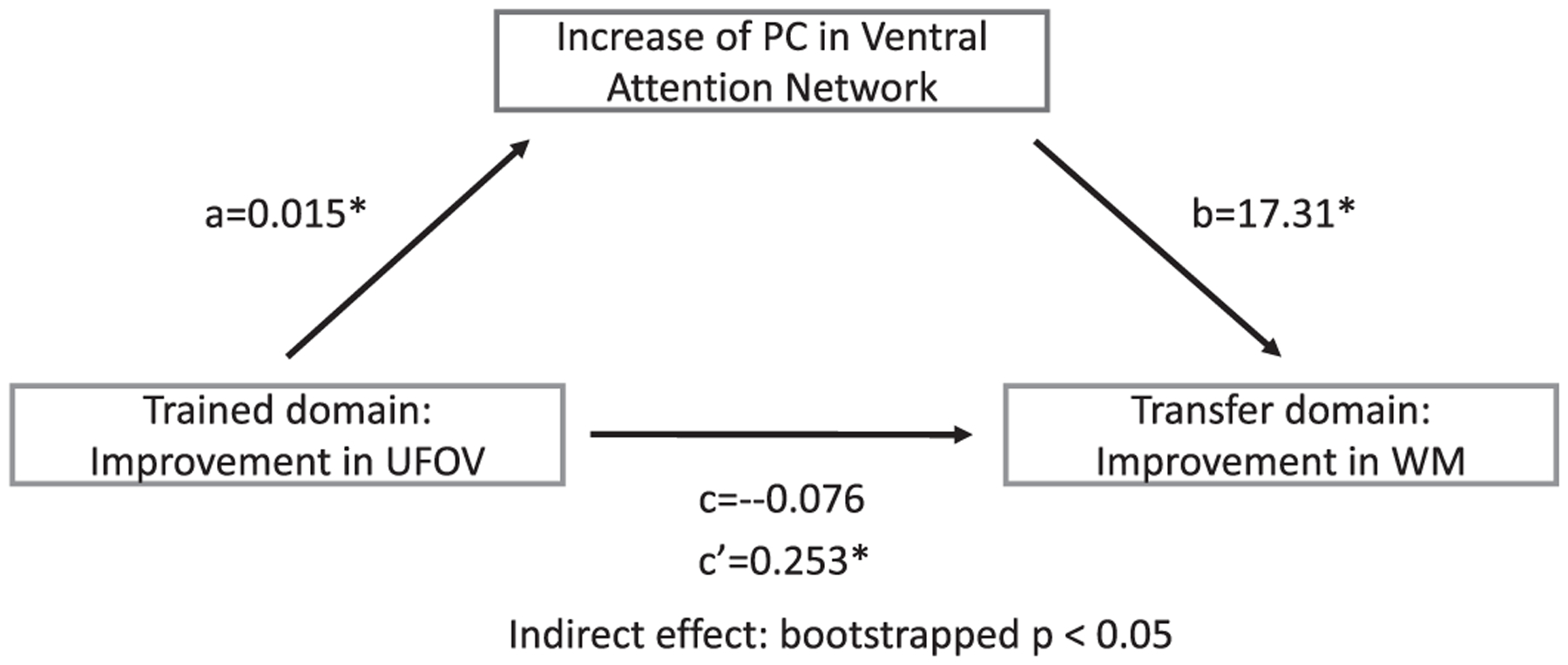
Increased PC in ventral attention network mediates transfer from the trained domain (i.e., UFOV) to the untrained domain (i.e., working memory). Mediation results are shown as standardized regression coefficients. C: total effect. C’: indirect effect. Significance of indirect effect was assessed using bootstrapped confidence intervals [0.0104–0.6091]. * indicates *p* < 0.05.

We didn’t find significant total effect (c) between UFOV and working memory (*p* = 0.816). Despite this insignificant total effect, mediation analyses revealed a significant indirect effect of ventral attention network PC (*p* < 0.05 based on 5000 bootstraps, 95%CI [0.0104, 0.6091], Fig. 6), suggesting that increase in PC score of ventral attention network mediated transfer from the trained domain (i.e., UFOV) to the untrained domain (i.e., working memory). No significant mediation effects were found for global PC or other networks.

Additionally, we tested whether neurodegeneration indexed by ADSCT (Cortical thickness signature for Alzheimer’s disease-associated neurodegeneration) affects PC. We didn’t find a significant effect of ADSCT on PC at global or network levels (all ps > 0.05). When controlling for ADSCT, the relationship of PC with VSOP-induced trained and transferred effects remained the same.

## Discussion

We demonstrated that increases in the PC of human cortical networks following VSOP training predicted improvement in an untrained working memory domain. This result was robust across global and network levels, with strongest effects observed in the ventral attention network. Critically, PC of the ventral attention network mediated transfer from the trained domain (i.e., UFOV) to the untrained domain (i.e., working memory). By using surface-based preprocessing and a novel pipeline (Cole et al., 2021) that projects the functional connectivity matrix to the white surface, we were able to capture the spatial features of the brain more accurately than using volumetric approaches (Brodoehl et al., 2020; Coalson et al., 2018), preserving spatial precision in our calculation of graphs that were used to calculate PC. This atlas-free approach provided us with 3661 × 3661 matrices for graph theory analysis, without the need to choose a fine-grained parcellation scheme, which can affect the topological properties of brain networks (Lord et al., 2016). To better interpret our final results and allow comparability with previous literature, nodal level PC that benefited from this improved precision and atlas-free approach was then averaged within large-scale networks that are widely used in the literature (Yeo et al., 2011).

These findings improve our understanding of cortical network reconfiguration in response to cognitive training and how the capacity for functional integration between networks contributes to successful transfer to complex cognitive abilities such as working memory. It has been hypothesized that successful transfer effects may be restricted to those domains that share common processing components and neural mechanisms (Jonides 2004; Lovden et al., 2011), and our results extend this idea by considering the neural processes underlying the integration of information between brain networks. While the trained (i.e., UFOV) and untrained (i.e., working memory) domains do share overlapping cognitive component processes, including visual perception, attention, and executive control, our results also suggest that the capacity for integrating information may represent a more subtle domain-general process that can be exploited to improve transfer effects to cognition more broadly. This is supported by the fact that the cortical networks that showed improved PC included many high-PC, or “diverse club ”, regions that are known to be particularly critical for supporting the integrative processing that supports complex cognition (Bertolero et al., 2017) such as working memory (Cohen and D’Esposito, 2016). Additionally, the fact that these results were identified at rest may suggest that they are more likely to generalize to trait-level cognitive functioning that shows some consistency between the laboratoy and the real world (Ho et al., 2020).

It is worth noting that VSOP training in our study did not lead to an increase in PC overall. Previous research has shown that UFOV training can induce strengthened functional connectivity between brain regions from ventral attention (e.g., anterior insula), default mode (e.g., anterior cingulate cortex), and frontoparietal (e.g., dorsolateral prefrontal cortex) networks (Ross et al., 2019) in healthy older adults in a manner that is consistent with increased PC. Additionally, in a previous study using the same sample of individuals as in our current study, we found that the same intervention did lead to increases in within-network functional connectivity (Lin et al., 2020), although between-network connectivity was not considered. These results suggest that VSOP training can alter resting brain organization in aMCI and PS/A training can improve between-network connectivity in healthy older adults. It may be that improving the capacity for integration via increasing between-network connections using these tasks is more challenging in individuals with aMCI, which would explain why we did not find a significant main effect in our study. This may also account for the fact that 51% of high-PC nodes were significantly activated by a similar PS/A task in our sample. Having identified improved integration as a mechanism for inducing transfer effects from simple visual attention tasks to more complex cognitive functions, a key goal of future research will be to understand how to develop tasks that target this neural mechanism in older adults, particularly those with aMCI. One innovative approach may be to adapt novel optimization procedures that can analyze fMRI data in real-time to modify task features to maximize a specific neural mechanism of interest (Lorenz et al., 2017). These approaches may be able to identify VSOP task features that are particularly able to activate networks known to be important for integration across multiple networks, leading to an increase in the ability of these task to activate a higher proportion of high-PC nodes/networks, or even to target features that increase the PC of these networks more specifically. The capacity for integration in the brain does appear to be modifiable in individuals at-risk for dementia via longer term pharmacological (de Waal et al., 2014) and more intensive cognitive training interventions (Dimitriadis et al., 2016), suggesting it may be amenable to cognitive training developed specifically for this purpose. These results also suggest that PC may be more easily influenced by non-VSOP training, and one important goal of future research will be to understand how these findings generalize across different types of cognitive training.

One important theoretical question involves how our findings relate to theories highlighting the importance of brain segregation, or modularity, for cognitive functioning (Cohen and D’Esposito, 2016). It has been proposed that showing distinct functionally specialized neural modules is a pre-requisite for the capacity to benefit from cognitive training due to plasticity (Gallen and D’Esposito 2019), and theories of aging suggest that in healthy older adults a loss of the modular structure of the brain may be responsible for cognitive aging (Koen and Rugg, 2019). Research has shown that the brain exists in a delicate balance of integration and segregation (Shine et al., 2018), neccesitated by the need to maximize the capacity for information transfer given limited biological resources to support wiring costs (Bassett and Bullmore, 2006). Functionally specialized modules are required for specific aspects of cognition, including motor execution, while other tasks, particularly those involving complex cognition requiring a range of different cognitive processes simultaneously (e.g., working memory) rely on integration (Cohen and D’Esposito, 2016). It may seem contradictory that training relies on both segregation and integration, however, it is clear that effective functioning requires both capacities, and both capacities appear to be at-risk from AD pathology, which can move the brain towards a more random network architecture without clear modules or the capacity for effective integration between them (Dai and He, 2014). Networks with high-PC nodes represent a key component of brain organization: having a relatively small number of nodes that are connected across all networks allows the brain to integrate information without compromising it’s modular structure (Bertolero et al., 2018; Bertolero et al., 2017). We propose that, while modularity is an essential characteristic of brain topology, integration via these networks is equally important, and that targeting PC as a mechanism of transfer exploits their unique role within the connectome to facilitate improved complex cognition without the risk of interfering with the inherent modular organization of the brain that is required for more specialized cognition. Therefore, while we chose to analyze PC due to the importance of diverse connections for integration during complex cognition, we understand that the connectome is a complex balance of integration and segregation that can be assessed using a range of graph theory metrics. The ability of the brain to form functionally specialized modules has been proposed as a critical biomarker for intervention-related plasticity, and networks/nodes with high PC have been shown to be critical for enabling brain modularity by allowing information to move between dissociated modules (Gallen and D’Esposito, 2019). Previous papers have also suggested that in some individuals, improving segregation as measured using clustering coefficient is also important for understanding responses to cognitive training (Chen et al., 2021), further highlighting that both integration and segregation metrics are important for intervention-related plasticity (Gallen and D’Esposito, 2019). While beyond the scope of this paper, future research is needed to clarify exactly how these different measures represent learning-dependent changes important for transfer, and to what extent these mechanisms are universal or dependent on baseline characteristics of individual connectomes.

There are several unanswered questions from our study: first, while we did find transfer effects related to improvements in working memory, we did not find any significant transfer effect relating to episodic memory. This may be explained by the fact that, relative to working memory, simple episodic memory retrieval as measured Brief Visuospatial Memory Test, does not place significant demands on cognitive control, which has been shown to modulate the need for neural integration (Ray et al., 2020). It will be important to test whether episodic memory performance in tasks that require more cognitive control and neural integration (Rosen et al., 2011) is more amenable to transfer via improved network integration. Alternatively, it may be that episodic memory impairments, which are more pronounced than working memory deficits in individuals with aMCI, are more intransigent to cognitive training. This would further increase the need to deploy cognitive training early on in cognitive decline, and motivates specialized approaches aiming to understand whether episodic memory can be improved by cognitive training in individuals with aMCI.

Second, we did not include subcortical regions in our analysis. Subcortical regions have been identified previously as important for transfer in younger adults due to their involvement in a range of cognitive processes (Dahlin et al., 2008). However, these regions also show damage early on in AD with significant consequences for functional connectivity (Son et al., 2017). As our hypothesis required transfer processes to be relatively preserved in aMCI, we chose not to include subcortical regions in our hypothesis. Additionally, determining how to calculate PC in subcortical regions is not straightforward. Many subcortical regions show connections with regions in every cortical network due to their roles in neuromodulation (Shine, 2019), and therefore deciding which functional module they should be located in is difficult. PC is also known to be affected by module size (Pedersen et al., 2020), meaning that if subcortical regions are treated independently they are likely to have a highly inflated PC as they have far fewer within-module connections than cortical networks. Alternatively, treating the subcortex as a single module carries its own conceptual issues, as this would require averaging across a range of functionally discrete regions leaving results difficult to interpret. Having considered these conceptual and methodological issues, we decided it would be clearer to focus purely on the cortical mechanisms underlying transfer. This does not diminish the potential role of subcortical connections in transfer, however, it may be that these regions are more important for modulating cortical integration than facilitating it directly (Shine, 2019). Future research specifically targeting the role of subcortical regions in the integration of information will be needed to further clarify these outstanding issues. Additionally, we chose to use the networks defined by Yeo et al. (2011) for this analysis due to their wide use in the literature and reliability in a large sample of individuals, allowing clearer comparison with the literature and greater reliability than using data-driven modules in our smaller sample. However, the use of these relatively large-scale networks limits the spatial specificity of our findings, meaning that it is unclear whether sub-networks within these networks may be driving results. Future research is needed aimed at understanding exactly how PC within these identified high-PC networks is important for transfer following cognitive training.

Finally, with enrollment starting in 2016, we followed 2011 NIH-AA diagnostic criteria and did not collect pathological data related to AD, as aMCI was considered a preclinical AD status. The revised 2018 NIH-AA research framework now recommends consideration of AD pathology; therefore, it is unclear whether VSOP training would be sensitive to individuals with positive AD pathology, and future studies including measures of pathology are needed to better understand the specific role of pathology in transfer effects.

## Method

### Ethics statement

The study was approved by the University of Rochester Research Subject Review Board. Written informed consented was obtained from each participant. All methods were performed in accordance with relevant guidelines and regulations. This study was registered with Clinicaltrials.gov on 24/09/2015 (NCT02559063).

### Participants

Eighty-four subjects diagnosed with aMCI (single- or multiple-domain) were recruited from University-affiliated memory, internal, and geriatric clinics. All clinics used 2011 diagnostic criteria for aMCI (Albert et al., 2011). Other eligible criteria have been described earlier (Lin et al., 2020). The study was approved by the University of Rochester Research Subject Review Board. All participants were sufficiently capable of providing an informed consent to participate on their own. Written informed consent was obtained from all participants. No adverse effects were associated with either intervention.

### Setting, randomization, and blindness

All assessments and selective intervention sessions were conducted in our research lab. Self-administered intervention sessions were conducted at participants’ homes or neighborhood community centers.

Intervention group assignment was designed using a 2:1 ratio in a 7-block randomization. Participants were notified by the interventionist about the intervention assignment (in a sealed envelope) only after all baseline data were collected. Outcome assessors remained blinded to the group assignment. Participants were informed of the comparison of two new computerized interventions throughout data collection to avoid the possibility of an uneven placebo effect. Participants who completed the MLA intervention were provided with a compensatory VSOP training at the end of the study.

### Interventions

Interventions were described previously (Lin et al., 2020). Briefly, VSOP training consisted of five tasks that emphasize PS/A, the task platform of which were provided by Posit Science (San Francisco, CA). All tasks share visual components that become increasingly more difficult and require faster reaction times as participants progress through the training. Participants responded either by identifying a specified target object or the location of the target on the screen. The training automatically adjusted the difficulty of each task based on the participant’s performance, thereby ensuring that participants consistently performed near their optimal capacity. Existing literature suggests that 12 h of cumulative VSOP training is sufficient to produce immediate as well as up to 11 months benefit in PS/A among cognitively healthy older adults (Ball et al., 2002; Wolinsky et al., 2013).

MLA training consisted of an online word search, Sudoku, and Free-Cell, a variation of solitaire. Participants were allowed to play any combination of these games to control for amount of computer use, and simulate to everyday mental activities.

Both interventions were conducted on online platforms specific to our study, with individual password-protected accounts. We provided all participants with an in-person training orientation and two in-person check-in sessions at our lab. All other training sessions were self-administered by the participants, with technical support available 7 days a week. The intervention lasted for six weeks, consisting of up to four 1-hour sessions per week. Participants’ access to both intervention platforms was removed on the date of their respective post-test assessments. No significant interaction effect between group and dose of intervention was found for changes in any variables.

Seven participants from the VSOP group, but none from the MLA group, dropped out during the intervention period due to non-study related reasons. Marital status was the only factor that approached significance in predicting dropout (57.1% of dropout subjects were unmarried vs. 23.4% among those that remained in the study, *χ*^2^ = 3.79, *p* = 0.052).

### Sample size estimation

Sample size calculations were performed using G*power. Based on parameter assumptions outlined in the protocol, the sample size (*N* = 84) provided 80% power to detect an improvement at Cohen’s *d* = 0.40, using two groups at the 2:1 ratio, and 4 repeated measures up to 6 months of follow-up with a 20% attrition rate.

### Measures

#### Cognitive measures.

PS/A was measured using UFOV, a three-task computer test that assesses processing speed, sustained attention, and divided attention based on reaction time. A composite score with natural log transformation was used, with higher scores indicating f reaction time and poorer performance (Ball et al., 1988). We inverted this score by multiplying it by −1, so that higher scores indicated better performance. Working memory was measured using a composite score derived from performance on dot-counting and dual-1-back tasks of EXAMINER, a computerized test battery, developed by NINDS and UCSF (Kramer et al., 2014). Episodic memory was measured using Brief Visuospatial Memory Test delayed recall T-score (Benedict et al., 1996). Across time points, different versions of the measures were administered to mitigate practice effects.

#### Imaging data

Imaging data were collected at University of Rochester using a 3T Siemens TrioTim scanner (Erlangen, Germany) equipped with a 32-channel head coil. **Structural MRI:** Each session began with a localizer scan, followed by an MPRAGE scan (TR/TE = 2530 ms/3.44 ms, TI = 1100 ms, FA = 7, 256 × 256 matrix, 1mm^3^ isotropic resolution, 1 mm slice thickness, 192 slices) to acquire high-resolution structural-weighted anatomical images. **BOLD fMRI** data were collected using a gradient echo-planar imaging sequence (TR/TE = 2500 ms/30 ms, FA = 90, 64 × 64 matrix, 4mm^3^ in-plane resolution, 4 mm slice thickness, 37 axial slices). Participants underwent a 5-minute resting-state scan, during which they were instructed to relax with their eyes open, followed by a 5-minute block-design “target among distractors” visual attention task (see also (Chen et al., 2020; Lin et al., 2020)). The stimuli were presented in 5 blocks, each of which consisted of 6 trials, for a total duration of 42 s; blocks were alternated with fixation periods of 20 s. Within each trial, a central fixation cross was presented for 500 ms, followed by 5500 ms presentation of the visual search pattern. An interval of 1000 ms was inserted between trials. Participants were instructed to search for the target symbol, “
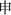
” (present for 50% of trials) displayed among 6 distractors in different orientations (e.g., “
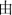
”, “
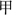
”.). Participants responded by pressing one of two response buttons to indicate whether the target was present or absent. We used a visual attention task because of its differences in task presentation compared to those of the VSOP training tasks while still containing a sustained attention component that is fundamental to performing VSOP tasks and for engaging in MLA.

#### Background information

Background information was collected at baseline. Cortical thickness signature for Alzheimer’s disease-associated neurodegeneration (ADSCT) was calculated using structural MRI data, with ADSCT ≤ 2.77 mm^3^ indicative of neurodegenerative atrophy (Jack et al., 2015; Lin et al., 2017). Dose of intervention was automatically counted by the VSOP and MLA training platforms. We also considered phenotype of MCI, single- vs. multi-domain, which was decided by their clinical diagnosis by the performance in executive function related battery tests. The analysis revealed no significant interaction effects between group and MCI phenotype for changes in any variables.

## Data analysis

### Imaging data preprocessing

Task fMRI data were analyzed with FEAT FSL Version 6.0.0 (www.fmrib.ox.ac.uk/fsl). Preprocessing of the functional data included: slice scan time correction (sinc interpolation), motion correction to the middle volume, smoothing with a nonlinear algorithm with 5 mm kernel, and high-pass temporal filter with sigma=100 s. For each participant, functional data were registered to high-resolution brain-extracted anatomical images in native space. Then functional and anatomical volumes were transformed into standardized MNI space. The general linear model (GLM) was used to fit beta estimates to the task events. The task events were convolved with a standard Double-Gamma hemodynamic response function. The six motion parameters were added to models as regressors. We assessed the task activation by the contrast of [task > rest]. The resulting statistical map was thresholded and corrected for multiple comparisons with FDR corrected *p* < 0.01.

For resting state fMRI data, we applied a Surface-Based Connectivity Integration (SBCI) pipeline (Cole et al., 2021). This surface-based pipeline was designed to enable the comparison of structural and functional connectivity, by projecting both signals to the white surface. T1 images were parcellated using FreeSurfer 6.0.0 (http://freesurfer.net/). Cortical surfaces were reconstructed using the recon_all tool available in Freesurfer. As in a previous paper (Cole et al., 2021), functional data was minimally preprocessed prior to being entered into this pipeline. Dicom images were converted to nifty format and the first 10 images of each scan were dropped to allow for stabilization of MRI signals. Images were then corrected for slice timing, and head motion using FSL MCFLIRT. Images were spatially smoothed in volume space with a Gaussian kernel with full width at half-maximum (FWHM) of 5 mm. Images were temporally filtered with a band-pass filter (0.01–0.08 Hz). The SBCI pipeline (https://github.com/sbci-brain/SBCI_Pipeline) was then used for further preprocessing using Freesurfer: motion correction, sampling to the surface (left and right), and surface smoothing with a Gaussian kernel with FWHM 5 mm. Nuisance covariates were regressed out, including the white matter signal, cerebrospinal fluid signal, six motion parameters (three translational and three rotational), and the global signal (Murphy and Fox, 2017), generating partial timeseries that controlled for these confounds. We mapped the volumetric BOLD signals to the participant’s cortical surface, resulting in a BOLD time series at each vertex on the surface meshes. The FC between any pair of vertices was calculated by correlating the two partial BOLD time series that had been controlled for confounding signals. This whole procedure generated a 3661-by-3661 symmetric weighted functional connectivity matrix per participant at baseline and post-intervention respectively.

### Network analysis

Each cortical vertex is assigned to one of 7 functional networks created by Yeo and colleagues (Yeo et al., 2011). The 7 networks consist of: visual, somatomotor, dorsal attention, ventral attention, limbic, frontoparietal and default mode networks (Fig. 1). Fig. 1 left shows the averaged fc matrix across all participants at baseline. We investigated the integration of brain networks at both the global and network levels. Following previous studies (Bertolero et al., 2017; Cohen and D’Esposito, 2016), and due to the fact that the interpretation of negative edges in FC networks is not clear, negative functional connectivity was set to 0. With the GRETNA toolbox (Wang et al., 2015), we calculated participation coefficient (PC) for each node, which measures how well a node within a given network is connected to other networks:

$$PC_{i}=1-\sum_{m=1}^{M}{\left(\frac{k_{i,m}}{k_{i}}\right)}^{2}$$

The term *k*_*i*,*m*_ denotes the sum of node i’s edge weight within module m, and *k*_*i*_ indicates the sum of node i’s edge weight in the entire brain. Nodes that interact with only nodes from its own module would have a PC of 0, while nodes with many distributed between-network connections would have a PC closer to 1. To estimate global network integration, we calculated the mean PC value across all nodes, which reflects the extent of integration between networks in the entire brain. To quantify the integration of specific modules, we calculated the mean PC value over nodes for each network, which reflects the extent to which a network connects to other networks.

### Statistical analyses

All statistical analyses were performed using SPSS version 24.0 (IBM).

#### Behavioral measures:

For behavioral measures, we calculated the group difference in reliable improvements, where a participant was classified as having improved reliably on a particular measure if their performance at a follow-up occasion exceeded baseline performance on that measure by 1 SEM of baseline data (cf., ACTIVE trial (Ball et al., 2002)). A Chi-square test was conducted with p-value set at 0.05. The effect size of training for each outcome was calculated using bias-corrected standardized mean difference (Hedge’s g): J*(M_training - M_control)/intra-subject standard deviation, where J is the bias-correction factor [1 – 3/(4*(total sample size-1) – 1)]ˆ(−1).

#### Relationships between behavioral and brain measures:

To test our hypothesis that higher functional integration of brain networks relates to greater transfer effects of cognitive intervention, we used Generalized Linear Model (*Change of Cognition* = *β*_0_ + *β*_1_ *Group* + *β*_2_ *Change of PC* + *β*_4_ *Change of PC* × *Group* + ԑ) at global and network levels. Significant between-group difference was based on the interaction of *Change of PC* × *Group*. Pearson’s correlation between changes of brain integration and behavioral variables were conducted for each group separately. One-tailed tests were used because our hypotheses were directional, expecting increased PC associated with greater transfer effects.

#### Main effect of training on brain measures:

Generalized Estimating Equation (GEE) model with AR(1) working matrix was used for between-group comparison with individual-level random effect considered: *y* = *β*_*0*_ + *β*_*1*_ *Visit* + *β*_*2*_ *Group* + *β*_*3*_ *Visit* × *Group* + ԑ*. Visit* was a later assessment (i.e. post-test) referred to baseline; *Group* was the VSOP group referred to MLA group; any significant between-group change was based on the interaction of *Visit* × *Group*.

#### Mediation analyses:

In order to determine whether brain integration could mediate the relationship between the trained domain (i.e., UFOV) and transfer domain (i.e., memory), we conducted mediation analyses with SPSS PROCESS Macro. Change of UFOV was the independent variable, change of PC was the mediator, and change of memory was the dependent variable. The PROCESS macro used bootstrapped confidence intervals to evaluate the significance of the indirect effect. Although traditional mediation analyses described by Baron and Kenny (Baron and Kenny, 1986) require a total effect to be present, it has been recently argued that the total effect shouldn’t be a prerequisite for tests of mediation (Hayes, 2009; Shrout and Bolger, 2002). For example, independent and dependent variables (X and Y) are fully mediated by two mediators, M1 and M2. The total effect is understood as the sum of the direct effect and all indirect effects. The total effect could be zero when the two indirect effects are comparable in magnitude but in opposite directions. In our case, it’s likely that other unstudied mediators carry the effect from trained domain through transferred domain in opposite directions, producing a total effect closer to 0.

#### Multiple Comparisons Control:

For network-level analysis, we adjust for multiple comparison across 7 networks with Benjamini–Hochberg (BH) procedure.

## Supplementary Material

1 Supplementary Figure. Group mean density for within- and between-networks (Tables SA-SI).

## Figures and Tables

**Table 1:** Baseline characteristics.

	Total (*N* = 84)	VSOP group (*n* = 56)	MLA group (*n* = 28)	t or *χ*^2^, df (p)
Age, mean (SD)	74.71 (7.30)	75.23 (7.49)	73.68 (6.92)	0.92, 82 (0.36)
Years of education, mean (SD)	16.34 (2.55)	16.17 (2.39)	16.68 (2.87)	−0.86, 82 (0.39)
Male, n (%)	45 (53.6)	32 (57.1)	13 (46.4)	0.86, 1 (0.35)
Non-Hispanic White, n (%)	74 (88.1)	52 (92.9)	22 (78.6)	3.63, 1 (0.06)
Married, n (%)	62 (73.8)	42 (75.0)	20 (71.4)	0.12, 1 (0.73)
MOCA, mean (SD)	24.05 (2.62)	23.89 (2.75)	24.36 (2.33)	−0.77, 82 (0.45)
GDS, mean (SD)	2.04 (2.23)	2.18 (2.15)	1.75 (2.40)	0.83, 82 (0.41)
Single-domain aMCI, n (%)	37 (44)	22 (39.3)	15 (53.6)	1.55, 1 (0.21)
First-degree family history of Alzheimer’s dementia, n (%)	43 (51.2)	28 (50.0)	15 (53.6)	0.10, 1 (0.76)
ADSCT in mm^3^, mean (SD)	2.77 (0.16)	2.75 (0.17)	2.81 (0.14)	−1.84, 82 (0.07)
Taking AD medication, n (%)	11 (13.1)	8 (14.3)	3 (10.7)	0.21, 1 (0.65)
BMI, mean (SD)	26.67 (4.64)	26.57 (4.75)	26.86 (4.50)	−0.27, 82 (0.79)
Chronic condition index, mean (SD)	4.46 (2.21)	4.38 (2.15)	4.64 (2.34)	−0.52, 82 (0.60)
• Hypertension, n (%)	45 (54.2)	27 (49.1)	18 (64.3)	1.73, 1 (0.19)
• Diabetes, n (%)	10 (11.9)	9 (16.1)	1 (3.6)	2.78, 1 (0.10)

NOTE: AD = Alzheimer’s disease; ADSCT = Alzheimer’s disease signature cortical thickness (for neurodegeneration); aMCI = amnestic mild cognitive impairment; BMI = body mass index; GDS = Geriatric Depression Scale – 15 items; MOCA = Montreal Cognitive Assessment (for global cognition).
