# Supplementary figures and images for: Enhancing cortical network-level participation coefficient as a potential mechanism for transfer in cognitive training in aMCI
Source: Neuroimage. Author manuscript; Available in PMC 2022 Jul 1. (PMC9199485; doi:10.1016/j.neuroimage.2022.119124)

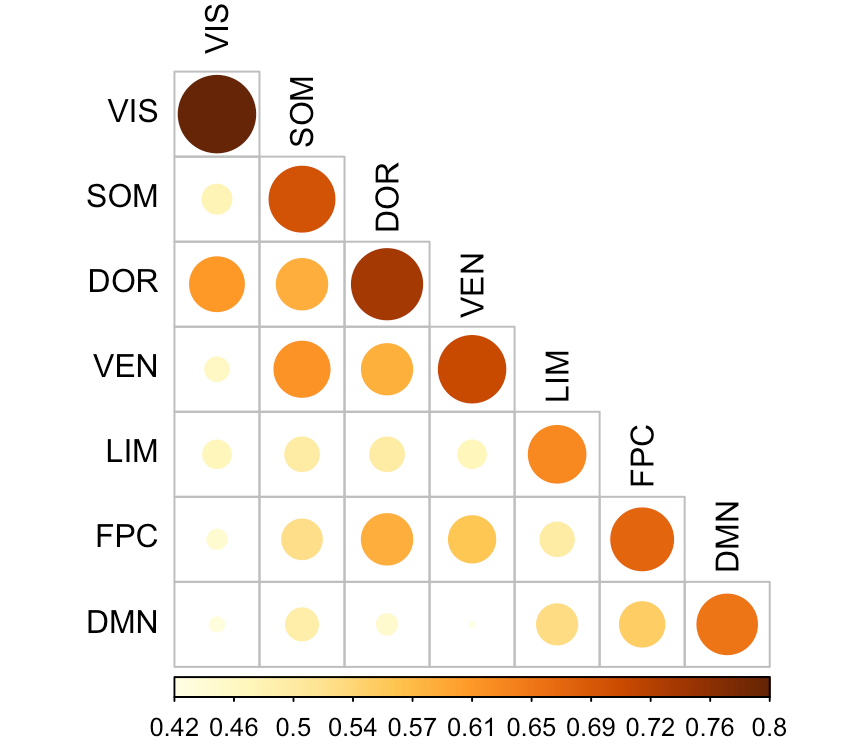


Supplementary Figure . Group mean density for within- and between-networks

Supplement: 1 — Supplementary Figure. Group mean density for within- and between-networks (Tables SA-SI). [file NIHMS1810853-supplement-1.docx]
